# Hereditary Hemochromatosis as a Potential Contributor to Ischemic Stroke: A Case Report

**DOI:** 10.7759/cureus.104077

**Published:** 2026-02-22

**Authors:** Mahsum Jafri, Zuhayr Khan, Constantino Lambroussis, Hunnan Jafri

**Affiliations:** 1 Internal Medicine, Lake Erie College of Osteopathic Medicine, Elmira, USA; 2 General Medicine, Lake Erie College of Osteopathic Medicine, Elmira, USA; 3 Osteopathic Manipulative Medicine, Lake Erie College of Osteopathic Medicine, Elmira, USA; 4 Department of Biology, Adelphi University, Garden City, USA

**Keywords:** atherosclerosis, carotid artery disease, cerebrovascular disease, endothelial dysfunction, ferritin, hemochromatosis, iron overload, ischemic stroke, oxidative stress, stroke risk

## Abstract

Hereditary hemochromatosis is a common inherited disorder of iron metabolism. It is classically associated with hepatic, cardiac, and endocrine complications. Neurologic manifestations, particularly ischemic stroke, are less commonly recognized and remain underrepresented in the literature. Our case focuses on a 54-year-old man with hereditary hemochromatosis who developed a subacute left pontine infarction in the absence of an identifiable cardioembolic source or large-vessel disease. Extensive evaluation failed to reveal a clear traditional etiology, prompting consideration of nontraditional risk factors. Given the patient’s history of iron overload, a hypercoagulable and microvascular mechanism was considered. Iron overload has been associated with endothelial dysfunction, oxidative stress, and prothrombotic changes, which may predispose a patient to ischemic events. Posterior circulation structures, supplied by small perforating vessels, may be particularly vulnerable to microvascular injury. This case highlights the potential role of hereditary hemochromatosis as an underrecognized contributor to ischemic stroke. Awareness of iron overload-related hypercoagulability may broaden the differential diagnosis in patients with cryptogenic or posterior circulation strokes. Further studies are needed to better define the relationship between iron metabolism and cerebrovascular risk.

## Introduction

Hereditary hemochromatosis is an inherited disorder of iron metabolism characterized by excessive intestinal iron absorption and progressive systemic iron overload [1]. Over time, excess iron accumulates in parenchymal tissues, leading to oxidative injury and dysfunction of multiple organ systems. The condition most commonly affects the liver, heart, pancreas, joints, and endocrine organs, with hepatic cirrhosis and cardiomyopathy among the most well-recognized complications [2]. Hereditary hemochromatosis is one of the most prevalent genetic disorders in individuals of Northern European ancestry [3], although clinical expression varies widely. While cardiovascular manifestations are well documented, cerebrovascular involvement remains comparatively underrecognized.

Neurologic complications of hereditary hemochromatosis are infrequently described in the literature. When present, reported manifestations more commonly include movement disorders or cognitive changes related to cerebral iron deposition rather than acute ischemic events [4]. Stroke, in particular, is rarely discussed outside of isolated case reports. As a result, the potential contribution of iron overload to cerebrovascular disease may be overlooked, especially when patients have concurrent traditional vascular risk factors. Emerging evidence suggests that iron excess may promote endothelial dysfunction, oxidative stress, and a prothrombotic state [5], providing a plausible mechanism for ischemic events that has not been fully explored.

Brainstem infarctions are especially concerning due to the compact anatomy and functional density of the posterior circulation, where even small lesions can result in profound neurologic deficits [6]. Recognition of atypical or systemic contributors is therefore critical in these cases. We present a case of brainstem infarction in a patient with hereditary hemochromatosis, highlighting iron overload as a potential contributor to a hypercoagulable state and ischemic stroke. This report adds to the limited literature on cerebrovascular complications of hereditary hemochromatosis and underscores the importance of considering iron overload in the evaluation of posterior circulation strokes.

We present a case of brainstem infarction in a patient with hereditary hemochromatosis, highlighting iron overload as a potential contributor to a hypercoagulable state and ischemic stroke.

## Case presentation

A 54-year-old Caucasian man with a past medical history significant for hereditary hemochromatosis with a homozygous C282Y mutation, JAK2-negative polycythemia without evidence of active erythrocytosis at presentation (hematocrit 47.1%), hypertension, hyperlipidemia, chronic low back pain with sciatica and lumbago, tubular adenoma of the rectum, diverticulosis, and hemorrhoids presented with acute neurologic symptoms. His hereditary hemochromatosis had previously been managed with regular therapeutic phlebotomy; however, he had not undergone phlebotomy for approximately two years prior to presentation.

The patient reported that his symptoms began while he was traveling and rushing, when he developed a posterior occipital headache that persisted for several days, which he described as unusual for him. During this time, he also noticed transient numbness of his gums but did not initially seek medical attention. After returning home, he became aware of worsening imbalance, followed by the development of right-sided facial droop, slurred speech, right-sided weakness, and ataxia, prompting hospital evaluation. He denied taking aspirin at baseline.

On arrival at the emergency department, his National Institutes of Health Stroke Scale (NIHSS) score was initially assessed as 4, with a subsequent evaluation yielding a score of 6. He remained hemodynamically stable but exhibited persistent neurologic deficits. Initial laboratory studies revealed a white blood cell count of 9.1 ×10⁹/L, hemoglobin of 16 g/dL, platelet count of 224 ×10⁹/L, potassium of 4.1 mmol/L, and creatinine of 1.4 mg/dL. Outpatient iron studies within the year prior to presentation demonstrated normal serum iron levels with decreased total iron-binding capacity and markedly elevated transferrin saturation of 86%. It also demonstrated mild hyperbilirubinemia and an elevated blood urea nitrogen-to-creatinine ratio.

**Figure 1:**
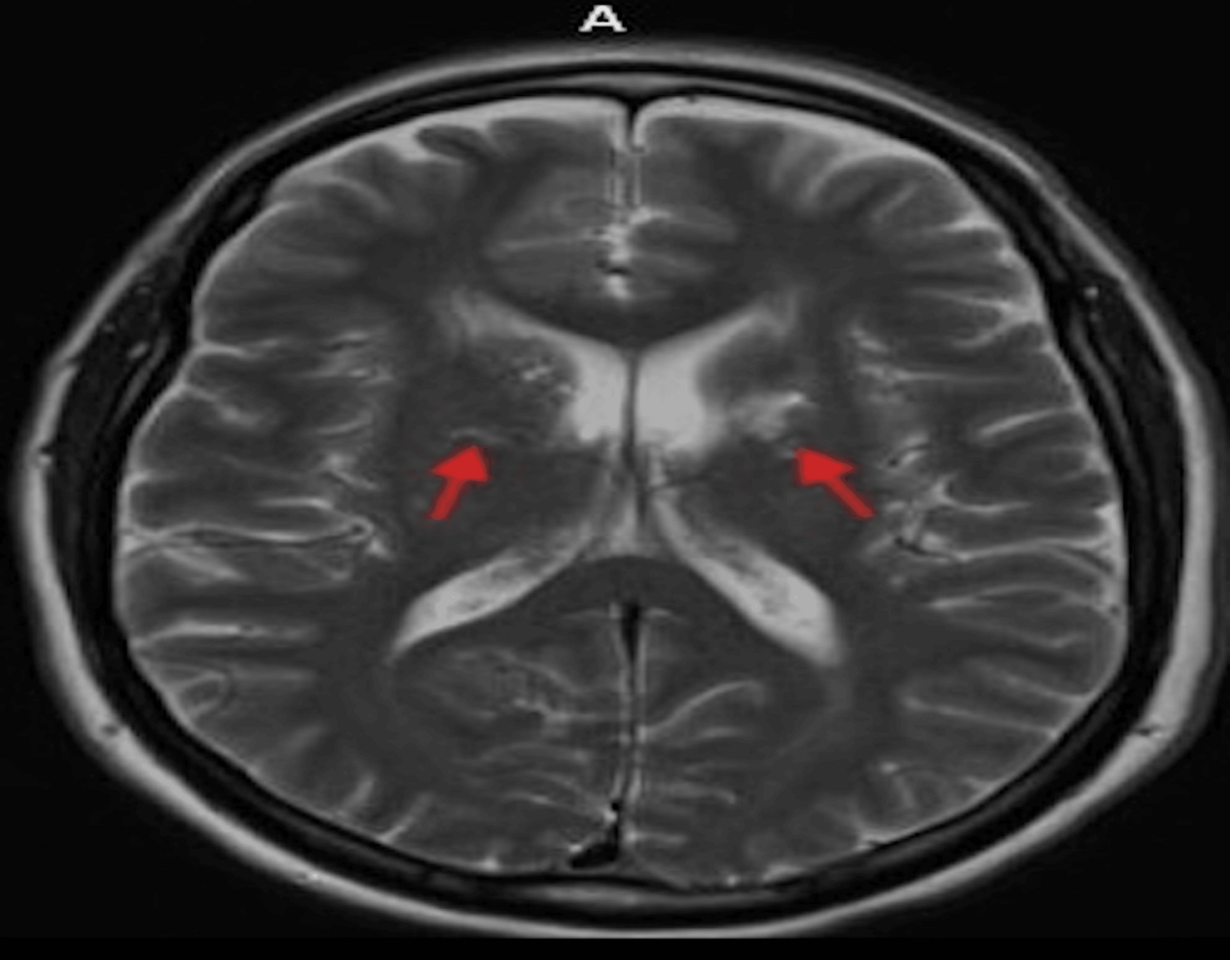
CT head showing chronic bilateral basal ganglia infarcts with microvascular changes.

**Figure 2:**
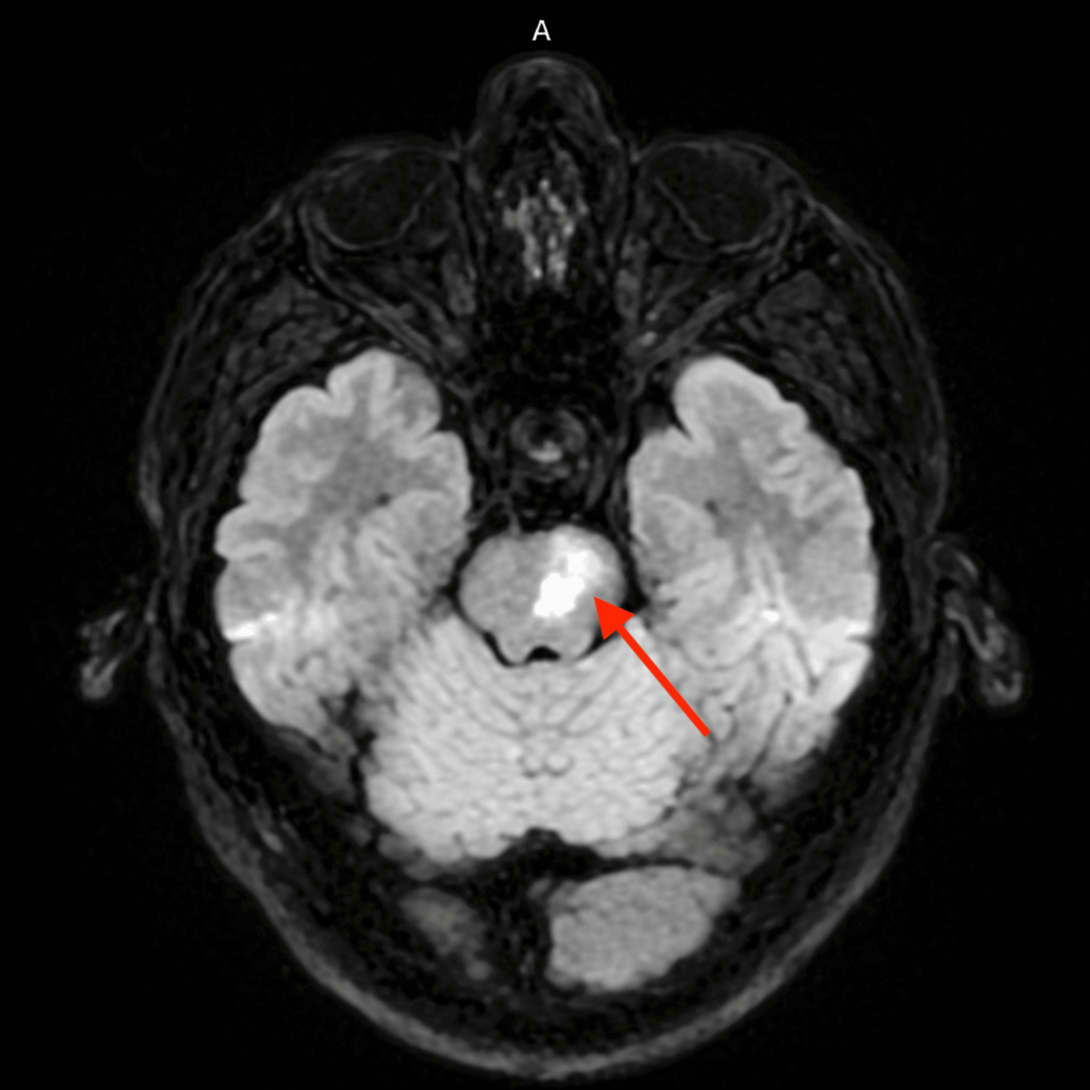
Brain MRI demonstrating a subacute infarct involving the left pons (arrow), corresponding to the patient's right-sided weakness, facial droop, and ataxia.

Neuroimaging with non-contrast computed tomography of the head demonstrated cerebral atrophy with chronic microvascular changes, including chronic-appearing bilateral basal ganglia infarcts (Figure 1). Computed tomography angiography of the head and neck showed no evidence of carotid or large-vessel stenosis. Magnetic resonance imaging of the brain revealed a subacute left pontine infarction with additional findings consistent with chronic microvascular ischemic disease (Figure 2). Transthoracic echocardiography demonstrated preserved left ventricular systolic function with an ejection fraction of 55-60% and no evidence of an atrial-level shunt. Continuous telemetry monitoring during hospitalization revealed no arrhythmias.

Teleneurology consultation recommended initiation of dual antiplatelet therapy. During hospitalization, the patient demonstrated subtle neurologic improvement without clinical deterioration. Antihypertensive medications were temporarily held to allow permissive hypertension. Additional laboratory evaluation showed a hemoglobin A1c of 5.1%, LDL cholesterol of 114 mg/dL, and a normal thyroid-stimulating hormone level.

The patient was evaluated by speech and physical therapy prior to discharge and was noted to have gradual improvement in strength and coordination. He was initiated on high-intensity statin therapy and dual antiplatelet therapy, with a plan to discontinue aspirin after 21 days and continue clopidogrel monotherapy thereafter. Antihypertensive medications were resumed following discharge. At discharge, his medication regimen included atorvastatin 80 mg daily, clopidogrel 75 mg daily, aspirin 81 mg daily for a planned duration of 21 days, losartan 100 mg daily, and hydrochlorothiazide 12.5 mg daily. Naproxen was discontinued. He was discharged on a cardiac diet with outpatient follow-up arranged. On follow-up, the patient reported continued functional improvement and had returned to work with ongoing physical therapy. He denied any history of tobacco use, including smoking or smokeless tobacco. He reported current alcohol use and denied illicit drug use.

## Discussion

Ischemic stroke in the absence of an identifiable cardioembolic source, large-vessel disease, or significant metabolic risk factors presents a diagnostic challenge and is often classified as cryptogenic. In our case, extensive evaluation failed to reveal atrial fibrillation, structural cardiac abnormalities, or large-vessel stenosis. The patient’s relatively young age and posterior circulation involvement further prompted consideration of nontraditional etiologies. When standard causes are excluded, systemic and hematologic contributors to cerebrovascular disease warrant closer evaluation.

Hereditary hemochromatosis represents a potential but underrecognized prothrombotic condition. Iron overload has been shown to promote endothelial dysfunction through oxidative stress mediated by reactive oxygen species, leading to impaired nitric oxide bioavailability and vascular injury [5]. Gaenzer et al. demonstrated that patients with hereditary hemochromatosis and elevated iron stores exhibited impaired endothelial function and increased carotid intima-media thickness, both of which correlated with markers of oxidative stress. Importantly, intensive phlebotomy resulted in significant improvement in endothelial function, supporting a causal role of iron overload in vascular dysfunction and thrombotic risk [7]. Population-based data further support a relationship between iron burden and vascular disease. In a large cohort study, higher serum ferritin levels were associated with increased carotid plaque prevalence, particularly among men, with evidence of a dose-response relationship. Notably, the association was potentiated by elevated LDL cholesterol, suggesting that iron may act as a vascular risk modifier rather than an isolated causative factor. While these findings primarily relate to atherosclerotic disease, they reinforce the broader concept that iron overload contributes to vascular pathology [8].

Endothelial injury facilitates platelet adhesion and activation, while iron has also been shown to directly enhance platelet aggregation and alter coagulation pathways [9]. Together, these processes create a proinflammatory and hypercoagulable state that may predispose to thrombosis even in the absence of markedly elevated serum iron levels at the time of presentation. Prior studies have linked elevated iron indices to increased risk of venous and arterial thrombosis, supporting a biologically plausible association between iron overload and ischemic stroke. 

Clinical evidence further supports a role for iron in ischemic stroke progression. In a prospective study of patients presenting with acute cerebral infarction, elevated plasma and cerebrospinal fluid ferritin levels within the first 24 hours were associated with early neurologic deterioration. Patients with higher iron indices demonstrated worsening neurologic scores despite similar initial presentations, suggesting that increased body iron stores may exacerbate ischemic injury through enhanced cytotoxic and oxidative mechanisms [10]. These findings support the biologic plausibility that iron overload contributes not only to stroke risk but also to stroke severity and progression. 

The location of the infarction in the brainstem further supports a microvascular mechanism. Posterior circulation structures are supplied by small perforating arteries with limited collateral flow, rendering them particularly vulnerable to endothelial injury and microvascular dysfunction [11]. Similar patterns are observed in other small vessel diseases, where systemic vascular pathology disproportionately affects deep brain structures. Iron-mediated oxidative stress may therefore preferentially impact these vulnerable vascular territories, contributing to ischemia in the posterior circulation.

Although neurologic complications of hereditary hemochromatosis are rarely reported, isolated case reports and small studies have described cerebrovascular events in patients with iron overload. These observations suggest that the neurologic manifestations of hemochromatosis may be underrecognized, particularly when traditional vascular risk factors coexist. Consideration of iron studies may be warranted in patients with cryptogenic stroke, posterior circulation infarcts, or concomitant hematologic abnormalities, as early identification and management of iron overload could potentially reduce thrombotic risk.

This case has important limitations. Iron studies, including ferritin and coagulation markers, were not obtained at the time of stroke, limiting assessment of iron burden during the acute event. Although the patient had a prior diagnosis of JAK2-negative polycythemia, hematocrit was normal at presentation (47.1%), with no evidence of active erythrocytosis, making hyperviscosity unlikely. Traditional vascular risk factors, including hypertension, mildly elevated LDL cholesterol, and chronic microvascular changes, may also have contributed. As a single case report, causation cannot be established; rather, this case suggests a biologically plausible association that deserves further study. 

## Conclusions

Our case highlights hereditary hemochromatosis as a potentially underrecognized contributor to ischemic stroke. When traditional causes are absent, iron overload may be considered among broader systemic factors in the differential diagnosis. Greater awareness of this possible association may encourage further investigation into the role of iron metabolism in cerebrovascular disease. Further studies are needed to clarify the relationship between iron metabolism and stroke risk and to determine how treatment strategies may influence long-term outcomes.
